# Proteomic analysis of nipple aspirate fluid to detect biologic markers of breast cancer

**DOI:** 10.1038/sj.bjc.6600285

**Published:** 2002-05-06

**Authors:** E R Sauter, W Zhu, X-J Fan, R P Wassell, I Chervoneva, GC Du Bois

**Affiliations:** Department of Surgery, Thomas Jefferson University, 1025 Walnut St., Ste 605, Philadelphia, PA 19107, USA; Department of Microbiology and Immunology, Kimmel Cancer Center of Thomas Jefferson University, Philadelphia, PA 19107, USA; Department of Biostatistics, Kimmel Cancer Center of Thomas Jefferson University, Philadelphia, PA 19107, USA

**Keywords:** proteomics, nipple aspirate fluid, breast cancer

## Abstract

The early detection of breast cancer is the best means to minimise disease-related mortality. Current screening techniques have limited sensitivity and specificity. Breast nipple aspirate fluid can be obtained noninvasively and contains proteins secreted from ductal and lobular epithelia. Nipple aspirate fluid proteins are breast specific and generally more concentrated than corresponding blood levels. Proteomic analysis of 1 μl of diluted nipple aspirate fluid over a 5–40 kDa range from 20 subjects with breast cancer and 13 with nondiseased breasts identified five differentially expressed proteins. The most sensitive and specific proteins were 6500 and 15 940 Da, found in 75–84% of samples from women with cancer but in only 0–9% of samples from normal women. These findings suggest that (1) differential expression of nipple aspirate fluid proteins exists between women with normal and diseased breasts, and (2) analysis of these proteins may predict the presence of breast cancer.

*British Journal of Cancer* (2002) **86**, 1440–1443. DOI: 10.1038/sj/bjc/6600285 www.bjcancer.com

© 2002 Cancer Research UK

## 

Standard screening for breast cancer involves physical examination and mammography. These tools alert the physician to the presence of a mass which is palpated and/or an abnormality which is visualised. Treatment of breast cancer requires a morphologic diagnosis characterised by visual changes in the nuclei in a cytologic or histologic preparation of breast cells. In order to obtain the cells for review by the pathologist, a diagnostic needle, core, or surgical biopsy must be performed. These procedures are painful, the needle and core biopsies are subject to sampling error, and only approximately 15–20% of the procedures detect malignancy (Watson *et al*, 1999). Non- and minimally invasive procedures are presently under review to detect breast cancer without submitting the subject to an invasive diagnostic procedure. These procedures include nipple aspiration, ductal lavage, and ductoscopy. Of these, only nipple aspiration is totally noninvasive and requires a device with minimal cost. In addition, only nipple aspiration provides concentrated secreted proteins undiluted with irrigation fluid which is required to perform ductal lavage and ductoscopy.

While cytologic and histologic evaluation are the gold standards to detect breast cancer, recent advances in comprehensive molecular technologies allow the simultaneous analysis of multiple protein expression targets. Two-dimensional polyacrylamide gel electrophoresis (2D-PAGE) and mass spectrometry have been used to perform proteomic analysis of human breast epithelium for comparison of cancer with normal breast (Czerwenka *et al*, 2001; Paweletz *et al*, 2001), changes in protein synthesis after growth factor induction (Vercoutter-Edouart *et al*, 2001), to detect new breast cancer markers and proteins involved in cell pathways such as signalling and the humoural response to antigens (Hondermarck *et al*, 2001; Le Naour *et al*, 2001), and to develop a breast cancer protein expression map database (Harris *et al*, 2002). Technologies in development include protein chips using antibodies or aptamers (short strings of DNA or RNA that bind with high affinity to specific target proteins) as capture molecules (Abbot, 2002).

A wide array of proteins are secreted into and highly concentrated in NAF and have been associated with breast cancer. We hypothesised that the proteome wide analysis of NAF using surface-enhanced laser desorption/ionization time of flight-mass spectrometry (SELDI-TOF) would identify one or more proteins differentially expressed in women with breast cancer compared to subjects without disease. The SELDI technique can be performed with 1 μl of NAF, can detect components in the high femtomole range, and the chip surface which allows the rapid evaluation of 8–24 samples has high throughput potential. Candidate breast cancer biomarkers can be identified using SELDI with a rapid immunoassay.

## MATERIALS AND METHODS

After informed consent of an Institutional Review Board approved protocol, NAF was collected using a modified breast pump (Sauter *et al*, 1996) and coded so that all analyses were blinded. Specimens were collected from women 35–81 years old scheduled to undergo surgery for a suspected malignancy in the aspirated breast, as well as from women without evidence of disease (Table 1

**Table 1:**
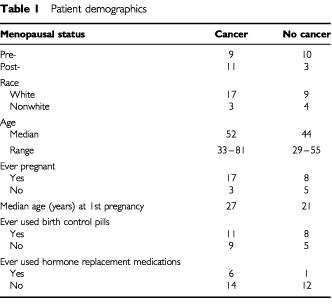
Patient demographics

). NAF was diluted with Tris buffer to a concentration of 3.6 mg total protein ml^−1^ Tris buffer. Proteome analysis with the SELDI PBSII system (Ciphergen Biosystems, Fremont, CA, USA) was carried out on three chips, normal phase (NP), hydrophobic (H4) and anion exchange (SAX), using 1 μl of NAF. The chromatographic surfaces of these ProteinChips allow the capture of generic proteins (NP), proteins having exposed hydrophobic surfaces (H4), and proteins binding by anion exchange (SAX). NP and SAX chips were washed first with binding buffer containing detergent, then with detergent-free buffer and water to remove nonspecific proteins. H4 chips were loaded with 10% acetonitrile, then washed with this solvent and water.

## RESULTS

**Figure 1:**
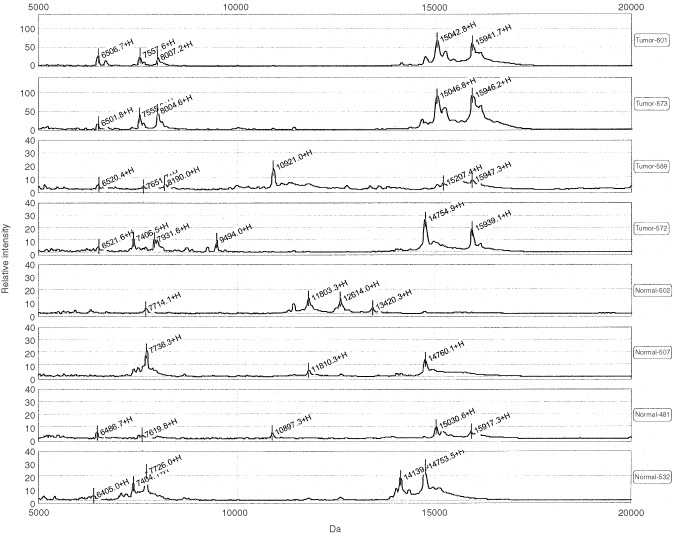
SELDI-TOF profile (5–20 kDa) from eight NAF samples. The top four lanes are from subjects with breast cancer.

A representative ProteinChip SELDI MS analysis of NAF demonstrates discrete peaks (Figure 1

) at 6500 and 8000 Da from three of four tumour bearing women which are not present or are present at a reduced level in NAF from women with normal breast tissue. We considered a protein expressed if the value assigned by SELDI software had a signal-to-noise ratio ⩾3 : 1. Protein peaks from different samples were judged similar if the values in Da were within 0.05% of each other. The figure also demonstrates a distinct peak at 15 940 Da in four out of four NAF samples from women with breast cancer that is not observed in NAF from normal subjects. Two larger proteins (a broad-based peak centered at 28 100 Da and a peak at 31 770, not shown) were also identified in a high percentage of women with breast cancer but were absent or present at a reduced level in NAF from normal women.

Percentages of positively expressed proteins in NAF from women with and without cancer and odds ratios (ORs) with 95% confidence intervals (CIs) are listed in Table 2

**Table 2:**
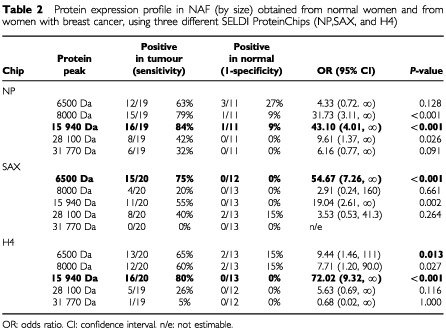
Protein expression profile in NAF (by size) obtained from normal women and from women with breast cancer, using three different SELDI ProteinChips (NP,SAX, and H4)

. Because of the small sample size, all estimates and *P*-values were computed using exact methods (LogXact 4.0, Cytel Software Corporation, Cambridge, MA, USA). Protein peaks at 15 940 Da in all chips, at 8000 Da in the H4 and NP chips, at 6500 Da in the H4 and SAX chips, and at 28 100 Da in the NP chip were expressed significantly more often in NAF samples from subjects with breast cancer than from normal subjects (Table 2). For each chip, maximum sensitivity and specificity were achieved with the single protein peak: 15 940 Da in the H4 and NP chips, and 6500 Da in the SAX chip (Table 2, bolded rows). These proteins were separately evaluated in multivariable logistic regression models controlling for age, race, past pregnancy, and past use of oral contraceptives. The adjusted odds ratios were: 29.2 (95% CI: 4.44 to ∞, *P*<0.001) for the 15 940 Da protein using the H4 chip, 27.3 (95% CI: 3.93 to ∞, *P*<0.001) for the 6500 Da using the SAX chip, and 18.7 (95% CI: 2.52 to 6.36, *P*=0.002) for the 15 940 Da using the NP chip.

## DISCUSSION

While both RNA and protein profiling can be applied to tissue samples, analysis of body fluids such as NAF is restricted to proteomics due to their low cellularity (Kennedy, 2001). Proteomic analysis of breast tissue has been reported using 2-D PAGE (Hondermarck *et al*, 2001). Although sensitive and powerful, 2-D PAGE is a labour intensive and low throughput method, has the drawback of selecting against proteins which are extremely acidic or basic, and there is a detection bias toward highly abundant proteins. The recently developed SELDI technique (Davies *et al*, 1999) allows for the rapid profiling of extracts from cells, tissues, and physiological fluids and can screen large numbers of samples in a clinical setting by differential protein capture according to chemical (ionic, hydrophobic, hydrophilic or metal ion affinity) surfaces on a protein chip.

Of the differentially expressed proteins identified using SELDI MS, the 6500 and 15 940 Da proteins are particularly striking since they were detected in a high percentage of subjects with breast cancer but in no one (6500 using the SAX chip and the 15 940 using the NP chip) or very few women without breast cancer. The identity of the 6500 Da protein is not known but may represent epithelin, while the 8000 Da peak may by mammaglobin. Mammaglobin has been reported to be a breast cancer marker (Watson *et al*, 1999). The identity of the 15 940 protein has not been determined. The 31 770 peak is most likely a dimer of the 15 940 protein.

The identity of the 28 100 Da peak may represent one or more members of the kallikrein family. A recent report using SELDI MS (Watkins *et al*, 2001) analysed the sera of 46 women with breast cancer and 23 controls. A 28.3 kDa protein was found in 100% of women with invasive and 80% with non-invasive breast cancer, respectively, but in only 4% of women without disease. This protein may correspond to the 28.1 kDa peak we observed in NAF, although confirmation is required. The putative breast cancer marker proteins that we have detected in this study may represent different isoforms of the candidate proteins or other known proteins. We are currently characterising the MS peaks identified in this study to determine their protein signature. We have also instituted a prospective analysis of NAF using SELDI-TOF to confirm the differential expression of these proteins in breast cancer *vs* normal breast, and plan to use a bioinformatics approach to identify the proteomic patterns in NAF that distinguish cancer from normal (Petricoin *et al*, 2002).

In conclusion, proteomic analysis of NAF from subjects with and without breast cancer identified five differentially expressed proteins. The most sensitive and specific proteins were 6500 and 15 940 Da, found in 75–84% of samples from women with cancer but in only 0–9% of samples from normal women. While current tools to detect breast cancer are widely available and clinically effective, they have limited sensitivity and specificity. Analysis of NAF proteins secreted from the breast epithelium may increase our ability to detect breast cancer at its earliest stages when used in combination with mammography and physical examination.
